# The association between labor companionship and obstetric violence during childbirth in health facilities in five facilities in the occupied Palestinian territory

**DOI:** 10.1186/s12884-023-05811-2

**Published:** 2023-08-05

**Authors:** Yasmeen Wahdan, Niveen M. E. Abu-Rmeileh

**Affiliations:** Institute of Community and Public Health, Birzeit University, West Bank Occupied Palestinian Territory, Birzeit, Palestine

**Keywords:** Labor,Companion, Mistreatment, Childbirth, Palestine

## Abstract

**Background:**

Studies show that the presence of companionship during childbirth leads to positive outcomes for women. This study investigates the protective effect of having a labor companion on obstetric violence in the health facilities of the Occupied Palestinian Territory.

**Methods:**

A secondary analysis of a cross-sectional study of women who gave birth in five health centers in the occupied Palestinian territory up to 8 weeks following childbirth was performed. The presence of a labor companion was examined in relation to socioeconomic variables and physical abuse, verbal abuse or stigma or discrimination, failure to achieve professional standards, vaginal examinations, and pain relief.

**Results:**

According to the findings, the total number of women with a labor partner or a birth companion present at any stage during the labor process was 92% in the West Bank, and 77.4% in the Gaza Strip**.** According to the timing of support**,** 23.5% of women had a labor companion present during labor, childbirth, and after childbirth whilst in the hospital. Women who did not have labor companions were more likely than women who did to report at least one sort of mistreatment, such as unconsented procedures. Women with a labor companion were less likely to report abuse (16%) compared to women without labor companion. In terms of informed permission for procedures, 75% of women who did not have a labor companion had unconsented episiotomy.

**Conclusion:**

Labor companionship assists women by providing them with companions who are less likely to be mistreated during labor. Efforts should be made to best implement the presence of labor companions, including the duration of the labor companionship and women's preferences.

## Introduction

Maternal health is defined as women's health during pregnancy, childbirth, and the postnatal period [1–3]. Each phase is an important event in the childbirth experience and should give women and their newborns to reach their recovery for health and well-being [3]. In global efforts to reduce maternal and newborn mortality and morbidity, the quality of care provided during childbirth in hospitals has been identified as a necessary factor. Global efforts to promote maternal health to advance the quality of care delivered to pregnant women [1]. Women's experience of care is a crucial aspect of the quality of treatment, as defined by the World Health Organization (WHO) as ensuring that all women are treated with respect, have excellent communication with health personnel, and have access to support that meets their conditions and needs [1–5].

The care experiences of women have a direct effect on their mental well-being. Consequently, cross-sectional research revealed that more than one-third of women were subjected to at least one form of mistreatment [2, 4]. Fifty percent of women in hospitals across four countries reported not having a labor companion [1]. Companionship during childbirth is defined as the continuous presence of a support person during labour and birth [4]. Therefore, having a labor companion there throughout birthing care is an excellent way to improve pregnant women's experiences by giving necessary care and support [2, 5]. Labor companionship improves the outcome of the woman and baby during childbirth [1, 6]. A Cochrane intervention review reported the significance of the presence of help for women during childbirth. Women with support were less likely to have a Caesarean section, less likely to report negative feelings about their childbirth care experience, labor was shorter, and there was a reduction in unpleasant birth experiences [7, 8].This companion can be the woman's spouse, husband, friend, healthcare professional, or family member [9, 10] According to the absence of a labor companion, women who did not have a labor partner were more likely to experience physical abuse, feel abandoned, and have poor communication [10].

Many countries do not yet have rules for labor companionship, and many healthcare facilities do not permit women to have a companion. So there are significant differences in hospital guidelines regarding labor companionship [1–3]. Private hospitals have adopted the presence of labor partners, although government hospitals have often disallowed this practice. This approach contrasts with women's demand for companionship or partners during labor and childbirth [4–6].

Despite labor companion importance, the presence of labor companions varies widely globally in different settings [8]. For example, 47.3% of women in Ghana have a labor companion [2]. 81% In brazil [4], 55% in Iran [11], 12.7% of women in Guinea, and 23% of women in Myanmar had a labor companion [2]. According to a multi-country survey, the most common person acting as labor companions were family members (mother/ mother in low) [3]. And 73% of Syrian women preferred to have their mothers with them during childbirth [8].

Also, if the women receiving care from the midwife, this support will reduced unplanned caesarean sections and other medical interventions during labour as well as reduced maternal and neonatal morbidity [12].

In the occupied Palestinian territory, mistreatment during childbirth was 18.8%; this study assessed the quality of care during childbirth identified mistreatment. The presence of a companion or absence will substantially affect the women through the childbirth process. With such quality of care, we would investigate the association of having a labor companion [10].

Few studies document the association between Labor Companionship and the effect on obstetric violence during Childbirth in the region, particularly Palestine. This study investigates whether there is an association between a companion's presence and quality of care, and the quality of care will be measured through mistreatment.

## Methods

This is a secondary data analysis based on a cross-sectional study done in the occupied Palestinian territory. A study started on 2/2019 until 2/2020 in five hospitals in West Bank and Gaza (three governmental hospitals, one private hospital, and one non-governmental hospital) [9]. In the preliminary investigation, five hospitals from the West Bank and Gaza Strip were chosen based on the inclusion criteria of (1) being a secondary or tertiary hospital and (2) having less than 200 births per month (3). Two hospitals were located in the heart of the West Bank, one public and the other private; two hospitals were located in the south, one public and the other private; and the final hospital was a public hospital in Gaza.

## Data collection

### Participants

Women were eligible to participate if they were at least 15 years old, had previously been admitted for childbirth, were willing to provide written consent to participate, were available for a follow-up interview up to 8 weeks post-partum, and lived within 15 km of the selected hospital [10].

Each study area had one data collector to trace the recruitment process. The data collectors were facility staff members from other departments that obtained appropriate training in safety measures and protection against COVID-19 compared to the research team [10]. For this study, they got clear instructions on the recruitment process and strategies, including the participants' wording, identification, eligibility lists, and reporting. The data collectors were trustworthy in accessing screening logs for hospital community surveys. Also, they documented to the participants that the interviews would be via telephone or video call, and the women had the choice to select the best method for them [10].

The data interviewers had to analyze the community survey screening logs in the hospital and decide if each woman was suitable for inclusion based on the criteria. The survey screening log was done by trained research assistants who were part of the research team, had been given adequate training in surveying and were not healthcare providers. Oral consent was obtained from women at the hospital to contact them within two to six weeks post-partum. Due to the COVID-19 lockdown and protective measures, we conducted phone interviews with women to decrease fieldworker mobility, as it posed an increased risk to the team and the community [10]. The interviewers got a second oral consent from women after they agreed to participate in the study and later conducted the telephone interviews. The interviewers attempted to contact women up to five times over a two-week. Eligible women whom we could not reach were called up to five times before reporting the loss offollow-up. The interviewers used a pilot study to obtain consistent results and improve the validity of the answers [10].

### Measurement tool

The development of the community survey tool has been described in previous studies [10]. Briefly, the questionnaire included different sections. The first one was related to sociodemographic data. The second was related to mistreatment typology (including physical and verbal abuse, stigma, or discrimination). The third section asked about the maternal outcome, babyoutcome, labor management practices, interventions, post-partum depression, and overall care satisfaction. An additional section related to assessing health service needs and provision during the COVID-19 pandemic was included. Finally, there were questions about the need and availability of health services, counseling services, and psychological services during the antenatal period and the need and availability of information about COVID-19, protective measures, and COVID-19 infection at the health facility during the childbirth process [10].

The primary independent variable in this study was the presence or absence of a labor companion at any time during care at the hospital, as reported by the women. (Total number of women with a labor partner or a birth-companion present at any stage during the labor process).

It was analyzed with dichotomous variables indicating the presence (yes/no) of any physical abuse (beating, slapping, kicking, pinching, or physically restraining women), nonverbal abuse (insulting, threatening, or blaming women), any stigma or discrimination (discrimination based on sociodemographic or medical characteristics), lack of informed consent during procedures (the procedure was not explained or permission was not granted), and poor communication (concerns were not listened or responded to). We also included a new indicator variable indicating of physical ubuse, verbal abuse, and stigma or discrimination [9]. Sociodemographic variables (age, education, marital status, number of pregnancies, number of previous births) and health outcome variables (physical abuse, verbal abuse, stigma or discrimination, failure to meet professional standards, vaginal examinations, and pain relief) were categorized as a proportion of the presence or absence of a labor companion.

### Data analysis

The characteristics of the labor companion, including the timing of support and the identity of the labor companion, were investigated using a simple descriptive analysis. In addition, the connections between sociodemographic variables, obstetric characteristics, and abuse type by the presence or absence of a labor companion were assessed by descriptive crosstabulation. This was achieved using varied grouping and categorization as detailed below:

The mistreatment typology, physical or verbal abuse sub-items and stigma or discrimination sub-items were aggregated into dichotomous variables (yes or no replies) and combined into a single indicator for each item.

The association between a companion's presence and mistreatment was evaluated using a bivariate approach (physical abuse; verbal abuse; stigma or discrimination; non-consented vaginal examination; non-private vaginal examination; neglect; long wait times or delays; and poor communication). The data was later analyzed with SPSS software.

## Result

The study involved 745 women, 475 from the West Bank and 270 from the Gaza Strip. Table 1 displays the characteristics of labor companionship by area (West Bank and The Gaza Strip). The presence of a labour companion at any point during care at the facility in oPt was as follow, 92.2 percent of women reported having a labor companion, compared to 96.7 percent in the Gaza Strip and 90 percent in the West Bank. In addition, 646 women reported the presence of a labor companion at any stage during the labor process. In the West Bank, 92% of women had a labor partner or birth companion present at any stage of labor, compared to 77.4% of women in the Gaza Strip. 23.5% of women had a labor companion present during labor, childbirth, and after childbirth in the hospital, ranging from 10% in Gaza to 35% in the West Bank, according to the time of support. Most labor companions were present during labor (75% in Gaza and 48.5% in the West Bank) and only after childbirth (10.2%). The most prevalent labor companions were family members (94.6%), followed by husbands/male partners (23.1%), and then presence of 2 labor companion (male partners and family members) (17.1%).

**Table 1:** Characteristics of labor companionship by region

	**Gaza *N* = 270 (%)**	**West bank *N* = 475(%)**	**Total *N* = 745 (%)**
**Total number of women with a laborpartner or a birth-companion present at any stage during thelabor process**	209(77.4%)	437(92.0%)	646(86.7%)
**Total number of women with a labor companion present at any point during care at the facility**	261(96.7%)	431(90.7%)	692(92.2%)
**Timing of support**			
Labor only	0	4(0.9%)	4 (0.5%)
Childbirth only	0	2(0.5%)	2 (0.3%)
After childbirth only	24(8.9%)	52(10.9%)	76(10.2%)
During labor and childbirth only	7(3.3%)	8(1.8%)	15(2.0%)
During labor and after childbirth only	157(75.1%)	212(48.5%)	369(49.5%)
During childbirth and after childbirth only	0	4(0.9%)	4(0.5%)
During labor, childbirth, and after childbirth	21(10.0%)	154(35.2%)	175(23.5%)
Don't know	0	1(0.2%)	1(0.1%)
**Labor companion**			
Husband/male partner only	14(6.7%)	135(30.9%)	149(23.1%)
Family member only	206(98.2%)	405(92.7%)	611(94.6%)
Both (male partner and family member)	12(5.7%)	103(23.6%)	115(17.8%)
Friend only	1(0.5%)	7(1.6%)	8(1.2%)
Women trained to help	0	0	0
Traditional birth attendants	0	0	0
Others	0	0	0

Table 2 presents Sociodemographic information and obstetric characteristics of women by labor companion status. So, women who reported no presence of a labor companion were aged less than 29 years 74.5%, and 37.8% had some secondary education; 30.0% had one previous birth, while29% this was their first pregnancy; 98% had delivered one baby in their most recent birth, and 71%had initiated breastfeeding within 24 h of birth.

**Table 2:** Sociodemographic information and obstetric characteristics of women by labor companion status

	No labor companion present	Labor companion present		
			**Total (*N* = 745)**	***p*-value**
**Maternal age (years)**				
≤ 19	14(14.3%)	78(12.1%)	92(12.4%)	0.367
20–24	29(29.6%)	150(23.2%)	179(24.1%)	
25–29	30(30.6%)	199(30.8%)	229(30.8%)	
30–34	13(13.3%)	135(20.9%)	148(19.9%)	
≥ 35	12(12.2%)	84(13.0%)	96(12.9%)	
**Marital status**				
Currently married	98(100%)	644(99.5%)	742(99.6%)	0.499
separated	0(0.0%)	3(0.5%)	3(0.4%)	
**Education**				
No education	1(1.0%)	0(0.0%)	1(0.1%)	0.005
Primary education	7(7.1%)	24(3.7%)	31(4.2%)	
Some secondary education	38(37.8%)	255(39.5%)	293(39.3%)	
Secondary education	15(15.3%)	72(11.1%)	87(11.7%)	
Tertiary education	38(37.8%)	291(45.0%)	328(44.1%)	
Vocational training	0(0.0%)	4(0.6%)	4(0.5%)	
**Number of pregnancies (gravidity)**				
1	28(28.6%)	149(23.1%)	178(23.9%)	0.488
2	16(16.3%)	143(22.1%)	159(21.4%)	
3	17(17.3%)	111(17.2%)	128(17.2%)	
4 +	37(37.8%)	243(37.6%)	280(37.6%)	
**Number of previous births (parity)**				
1	31(30.6%)	169(26.3%)	200(27.0%)	0.733
2	21(21.4%)	146(22.7%)	167(22.5%)	
3	15(15.3%)	122(19.0%)	137(18.5%)	
4 +	32(32.7%)	206(32.0%)	238(32.1%)	
**Breastfeeding**				
Currently breastfeeding†(yes)	97(99.0%)	596(92.1)	693(92.2%)	0.005
Currently breastfeeding†(no)	1(1.0%)	51(7.9%)	52(7.8%)	
**Breastfeeding initiation†**				
Within 1 h	70(71.4%)	401(64.6%)	471(65.5%)	0.234
Within 24 h	24(24.5%)	167(26.9%)	191(26.6%)	
Within 1 week or longer	4(4.1%)	53(8.5%)	57(7.9%)	
**Number of babies at most recent birth**				
One baby	96(98.0%)	625(96.7%)	721(96.9%)	0.397
Two babies	2(2.0%)	21(3.3%)	23(3.1%)	

Table 3 presents the prevalence of mistreatment typology by presence or absence of a labor companion. Women with a labor companion were significantly less likely to report any type of abuse (16%). More specifically, women with a labor companion were less exposed to physical abuse (2.5%) compered to (8.2%) of women without labor companion, and verbal abuse (14.7%) compared to (28.6%) of women with no labor companion.

**Table 3:** Mistreatment among women with and without a labor companion present at any point during care

	No labor companion present	Labor companion present	**Total**	***p*-value**
**Any physical abuse, verbal abuse, or stigma, or discrimination**	34(34.7%)	105(16.3%)	139(18.7%)	0.000
Any physical abuse	8(8.2%)	16(2.5%)	24 (3.2%)	0.008
Any verbal abuse	28(28.6%)	95 (14.7%)	124(16.6)	0.001
Any stigma or discrimination	0(0.0%)	5(0.8%)	5(0.7%)	0.494
**Failure to meet professional standards**				
Caesarean section (*N* = 745)	23(23.5%)	180(27.9%)	203(27.3%)	0.217
Unconsented Caesarean section (*N* = 203)	3(13.1%)	62(34.4%)	65(32.0%)	0.127
Episiotomy‡ (*N* = 251)	32(43.2%)	219(51.1%)	251(33.6%)	0.130
Unconsented episiotomy (*N* = 251)	24(75.0%)	152(69.4%)	176(70.1%)	0.001
Induction of labor (*N* = 700)	17(20.5)	123(20.0%)	140(20%)	0.521
Unconsented induction of labor (*N* = 140)	15(88.2)	102(82.9%)	117(83.5%)	0.177
**Vaginal examination**				
Woman had any vaginal examination	89(90.8%)	540(83.6%)	630(84.4%)	0.040
Unconsented vaginal examination	57(64.0%)	319(59.1%)	376(59.7%)	0.222
**Pain relief**				
Woman requested pain relief	39(39.8%)	345(53.3%)	384(51.5%)	0.008
Woman requested pain relief but did not receive pain relief	19 (48.7%)	168(48.6%)	187(48.6%)	0.002
Woman denied pain relief during time in hospital	26(27.4%)	86(13.4%)	112(15.2%)	0.001
**Neglect and abandonment**				
No staff member present when the baby came out ‡	7(9.6%)	48(10.5%)	55(10.4%)	0.506
Woman waited along time before attended by health workers	30(30.6%)	213(32.9%)	243(32.6%)	0.367
Woman felt ignored, neglected, or that her presence was a nuisance for health workers or staff	36(36.7%)	137(21.2%)	173(23.3%)	0.001
**Communication**				
Woman felt that health workers or staff not listened and responded to her concerns	44(45.4%)	444(70.0%)	488(66.7%)	0.000

Regarding informed consent for procedures, 75% of women without a labor companion received unconsented episiotomy. There was no significant difference in the prevalence of reported consent for vaginal examinationcare, which was 64% without a labor companion episiotomies (75% without a labor companion and induction of labor (88% without a labor companion).

Regarding pain relief, there was a significant difference in terms of women who requested pain relief between women with a labor companion 53%compared to those without a labor companion40%. Further, the percentage of womenwho denied pain relief during their time in the hospital was 27% for women without a labor companion.

According to communication, women with a labor companion were more likely to report that health staff not listened or respond to their concerns (70%) compared to women without alabor companion (45%). However, women with a companion had no significant difference in waiting a longtime before being attended by health workers (32%) compared to women without a companion (30%). In terms of the absence of a staff member when the baby came out,there was no difference between women with a labor companion (10.5%) and women without a labor companion (9.6%).

## Discussion

We presented the results of a community-based survey in the Occupied Palestinian Territory. We outlined the characteristics of labor companionship and investigated the relationship between labor companionship and abuse experiences. Women without labor partners were more likely to report mistreatment, and unconsented episiotomy than those with labor companions. Women with a labor partner were substantially less likely (16%) to report abuse. In general, women who had a companion to be present throughout labor and childbirth were less exposed to feelings of Neglect and ignore from health staff during childbirth. This was deemed a key aspect in boosting the quality of treatment during labor and childbirth [13, 14].

The findings showed that the most common labor companions were mothers and mothers in lows (family members), then the husband. This is related to cultural reasons, as the presence of a male companion is frowned upon in many societies. As a result of the fearful form effect of the birthing scene on the sexual relationship.Or it was possibly owing to structural issues such as the presence of many women in the same room [1]. Labour companion is family or friends but the other type of support, such as trained women during childbirth (Doula (s), traditional birth attendants), was non-existent. But it is available in another context. We could not find it in the Palestinian context —moreover, the absence of this trend in Palestinian society. Because of Doula's support may be specifically applicable to perinatal immigrant women, especially those with limited resources, lack economic resources, and cannot access the best care and treatment [7, 14, 15].

Our finding reported the differences between Gaza and the West bank in having family members vs. having a husband, and this is due to cultural issues such as the bonding between the male–female relationship is strong. Our findings reported differences in having family members vs. having a husband between Gaza and the West Bank, and this is due to cultural issues (17–18) or hospital policies because the hospital in Gaza that collected the data was a governmental hospital where structured labor wards might not support companionship during childbirth [14].

Women who did not have a labor companion were more likely to have a bad birth experience [7, 16]. One of the most important advantages of the present companion is that it can lessen women's vulnerability to abuse [7, 17, 18]. This result is shown in the results, as 16.3% of women with a companion reported experiencing at least one sort of abuse, compared to 34.7% of women without a companion. This finding is in line with previous research indicating that the absence of a labor companion increases the risk that a woman will experience mistreatment during childbirth and that the presence of a labor companion may protect against mistreatment and lead to a positive birth experience for women [1]. Also, based on the findings of the studies, the absence of a labor companion decreased the mothers' ability to express that they experience conflicts in various spheres of their daily lives and cannot discuss them with anyone. One of the main findings was that women with a labor companion were less exposed to unconsented vaginal examination, and unconsented episiotomy, which is consistent with evidence indicating that the presence of a labor companion decreased the possibility of unconsented treatments [10]. This finding is consistent with a review of women's perspectives conducted in high-income countries since labor companions can act as advocates who increase the communication of women's preferences to health workers [10].

In our study, the percentage of women who requested pain relief was higher among women with labor companions. This result differs from the previous evidence, which indicates that the presence of a labor companion makes women request less pain relief [7, 10, 15]. However, the explanation can be that the presence of a labor companion increased her ability to ask for pain relief [19, 20] Companions were supporters, which indicates they spoke up in support of the woman.Companions delivered helpful service, encouraging women to ask and discuss the health staff [16, 21].

Regarding communication, women with a labor companion reported feeling that health workers or staff listened and responded to their concerns. Also, the results indicated that they were less likely to feel ignored or neglected or that their presence was a nuisance to health workers or staff. This aligns with other studies that indicate that labor companions can be intermediaries who enhance the communication of women's preferences to clinical staff [1, 10, 11]. Also, companions can facilitate the communication process by helping women to feel in control of their concerns [2, 3, 12].

This study was conducted during the COVID-19 pandemic. A global study reported that the COVID-19 pandemic has negatively affected the provision of health care and women's right [22]. fortunately, although the health system in the oPt was struggling with the pandemic, the presence of labor companions in the Palestinian context was high considering the provision of essential reproductive and maternal health services during the pandemic [23–26].

## Conclusion

The presence of a labor companion was associated with a reduced risk of physical abuse, unconsented episiotomy, and less exposure to feeling ignored by medical professionals. Allowing women in occupied Palestinian territory to have a birthing companion of their choice can be a low-cost and effective method for reducing violence against women during labor and delivery. In the Palestinian context, efforts should be taken to adopt labour companionship across several elements and to guarantee that women's autonomy and choice are respected.

### Strengths and limitations

This study documented relationship between the presence of labor companions and abuse during childbirth in Palestine especially durcing the COVID-19 pandemic when hospitals were required to prevent infections and maintain a suitable physical distance. However, the study analyzes secondary data and labour companion was secondary outcome and hence several factors of importance, such as the duration of a companion's presence, were missing.

## Data Availability

The datasets used or analyzed during the current study are available from the corresponding author upon reasonable request.
